# Prognostic value and immune infiltration of anoikis-related genes in osteosarcoma

**DOI:** 10.3389/fmed.2025.1669470

**Published:** 2025-11-06

**Authors:** Jianming Mo, Hening Li, Hui Wang, Xu Fang, Jie Ma, Kaiwei Chen

**Affiliations:** 1 Department of Orthopaedics, Minzu Hospital of Guangxi Zhuang Autonomous Region, Nanning, Guangxi, China; 2 Department of Oncology, The First Affiliated Hospital of Guangxi Medical University, Nanning, Guangxi, China; 3 Department of Spinal Surgery, Liuzhou Workers Hospital, The Fourth Affiliated Hospital of Guangxi Medical University, Liuzhou, Guangxi, China

**Keywords:** osteosarcoma, prognosis, single-cell RNA sequencing, anoikis, immune infiltration

## Abstract

**Background:**

Currently, there is no research on building osteosarcoma (OS) prognostic models based on single-cell RNA sequencing (scRNA-seq) and anoikis-related genes (ARGs).

**Methods:**

Differential genes between osteoblasts cells and osteosarcoma cells were identified using scRNA-seq, and ARGs were determined by Genecard database. Lasso regression was employed to investigate hub genes and construct the model based on TARGET. Kaplan-Meier survival analysis was applied to compare the survival differences. ROC curves were used to evaluate the predictive performance of the model. CIBERSORT and ESTIMATE algorithms were conducted to calculate immune cell infiltration abundance. Finally, qRT-PCR and immunohistochemistry experiments were conducted to validate the results.

**Results:**

A predictive model containing four modeling genes (MYC, BNIP3, IGFBP5, and SPP1) was successfully constructed, with AUC values of 0.836, 0.837, and 0.836 for 1-, 3-, and 5-year patient prognosis, respectively. Importantly, the model also showed good predictive value in two validation set. The infiltration of immune cells in different risk groups showed significant differences. The modeling genes were associated with the expression of various immune checkpoints and the response to immune therapy. qRT-PCR showed MYC, BNIP3, IGFBP5, and SPP1 substantially exhibited a trend of high expression in osteosarcoma cells. Immunohistochemistry suggested that in osteosarcoma patients with poorer prognosis, the expression of these four hub genes was significantly elevated.

**Conclusions:**

We have developed an effective model for predicting osteosarcoma prognosis and immune response, which may provide valuable insights for osteosarcoma prognostic evaluation and immune therapy strategies.

## Introduction

Osteosarcoma (OS) is a primary malignant bone tumor originating from mesenchymal tissue and commonly affects children and adolescents, exhibiting a highly aggressive nature. Despite improvements in therapeutic approaches for OS, the 5-year survival rate remains at around 60% (1). In recent years, many researchers have focused on constructing prognostic prediction models for OS based on clinical and pathological features, biochemical tests, and molecular characteristics of tumor tissues (2). However, these models have certain limitations and are currently not applicable for clinical practice. Therefore, there is still a need to supplement and refine prognostic model.

Anoikis, a form of programmed cell death, refers to the process where normal adherent cells will detach from the extracellular matrix (ECM), fail to survive in a suspended state, and ultimately undergo cell death due to the lack of anchorage support (3). Under physiological conditions, anoikis will be activated after losing attachment, which play a key role in maintaining normal tissue structure. However, in certain pathological conditions, such as cancer, anoikis can be inhibited by cells. In processes such as tumor invasion and metastasis, tumor cells detach from the primary site, invade and implant into secondary sites through the lymphatic and circulatory systems, and then proliferate and grow, making resistance to anoikis the initial step in tumor invasion and metastasis (4). It has been found that most tumor cells possess resistance to anoikis, releasing its crucial factor in promoting tumor cell survival, invasion, and metastasis (5). Meanwhile, given the increasing significance of immunotherapy as a major treatment for cancer, the regulatory role of anoikis in immunity has drawn attention in various types of tumors (6). However, the relationship between anoikis and the immune microenvironment in OS, as well as its impact on prognosis, remains relatively understudied.

In this study, we employed single-cell sequencing (scRNA-seq) and bulk RNA sequencing (bulk RNA-seq) technologies to develop a prognostic model for OS based on anoikis related genes (ARGs). The model was validated using two external dataset. Additionally, the prognostic value of the model genes was assessed through PCR and immunohistochemistry experiments. On one hand, the hub genes identified in this study not only promote the onset and progression of OS but are also related to anoikis. This provides targets for further investigation into the mechanisms of anoikis in OS. On the other hand, the model demonstrates strong predictive efficacy and robustness, laying a foundation for improving the prognosis of OS patients.

## Methods and materials

### Flow chart

**Figure 1:**
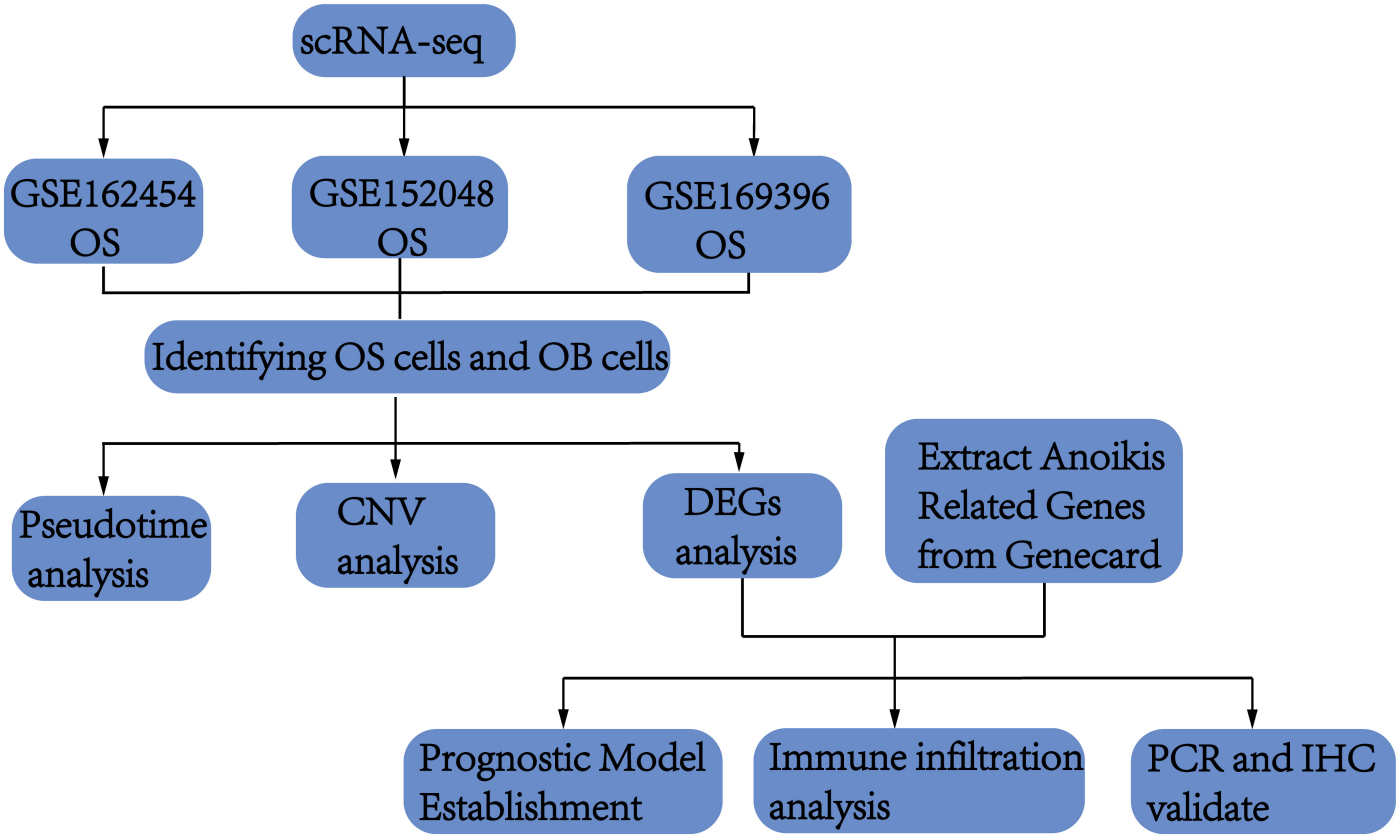
A flowchart of the process outlining the analysis pipeline for osteosarcoma scRNA-seq.

This study initially collected scRNA-seq data from OS and normal bone tissues to identified osteoblasts. Differential analysis of osteoblasts from both tissues revealed key genes potentially involved in OS pathogenesis. These genes were then intersected with ARGs, which are implicated in promoting OS through anoikis mechanisms. Using both scRNA-seq and bulk sequencing data, we constructed and validated a prognostic model. Finally, the identified genes were validated through cell experiments and immunohistochemistry. A flowchart of the process was shown in Figure 1.

### Data sources

The single-cell RNA sequencing data included four femoral head tissue samples from the GSE169396 dataset in Gene Expression Omnibus (GEO), six OS tissue samples from the GSE162454, and seven OS tissue samples from GSE152048. In the preliminary experiments, tumor tissues collected during surgery were cut into approximately 1 mm^3^ fragments and converted into cell suspensions for further use. The data were sequenced in paired-end mode, measuring 150 bases on both Read 1 and Read 2 ends. The upstream analysis of the data was performed using Cell Ranger software (version 4.0.0) following the official 10 × Genomics workflow. The single-cell data were aligned to the human genome reference library GRCh38. Barcode.tsv files, gene.tsv files, and matrix.mtx files were obtained through paired read length, feature barcoding, clustering, and other secondary analyses.

Additionally, bulk sequencing data was included to build the predictive model. A total of 85 OS samples from the TARGET database were used as the experimental dataset for model construction, and the dataset GSE21257 (53 OS patients) and GSE16091 (34 OS patients) downloaded from the GEO was used for model performance validation.

### Identification of ARGs

A search with the keyword “anoikis” was conducted in the genecards database (https://genealacart.genecards.org/), and genes with a relevance score >0 were included in this study.

### Identification of OS cell markers

In the working environment formed by the “Seurat” and “harmony” packages, scRNA-seq data were used to screen for OS cell markers. Criteria were set as (nFeature_RNA >300, nFeature_RNA < 4,500, percent.mt < 15) to rigorously filter cells and identify OS cells. The data of filtered cells were normalized using the “NormalizeData” function and transformed into Seurat objects. The top 2,000 highly variable genes were subjected to dimensionality reduction using the “RunPCA” function, and the top 20 principal components (PCs) were determined by JackStraw analysis. The “RunHarmony” function from the “harmony” R package was used to remove batch effects. Subsequently, cell clustering analysis was performed using the “FindNeighbors” and “FindClusters” functions in the “Seurat” R package, and the clustering results were visualized using the “RunUMAP” function to generate Uniform Manifold Approximation and Projection (UMAP) plots. The “FindAllMarkers” function of the “Seurat” R package identified differentially expressed genes (DEGs) for each cluster based on the criteria of adjusted *P* < 0.05 and |log_2_(FC)| >0.25. The DEGs of the OS cell cluster were extracted as cell markers.

### Establishment of the prognostic model and risk scoring related to anoikis genes

The overlap between the OS markers and the ARGs, which is also called OS ARGs formed the research object. The transcriptional profiles of ARGs were obtained from the transcriptional dataset of 85 OS samples from the TARGET database. Additionally, LASSO regression analysis, as one of the machine learning algorithms, was used to select genes with prognostic significance. Therefore, the expression profiles of ARGs were combined with clinical information, and the “cv.glmnet” function of the “glmnet” R package was used for 10-fold cross-validation to obtain nonzero coefficients for prognostic genes. These prognostic genes constituted a prognostic model named OAPM. The formula for OAPM was defined as follows: risk score = Σexpgenei ^*^ βi, where “expgene” and “β” represent the expression level and risk coefficient of the model genes, respectively.

### Validation of the prognostic model OAPM

The prognostic model was validated in multiple datasets to demonstrate its excellent predictive performance. The experimental dataset from the TARGET cohort and the validation dataset GSE21257 and GSE16091 were divided into two groups based on the median risk score. The “survivalROC” R package was used to obtain time-dependent ROC curves, and the area under the curve (AUC) values reflected the predictive ability of OAPM for 1-, 3-, and 5-year overall survival of the patients. Kaplan-Meier survival analysis was conducted using the “survminer” R package to investigate the survival differences between the high-risk and low-risk groups. Additionally, independent prognostic analysis was performed to test the potential of OAPM as an independent prognostic factor. Clinical forest plots were constructed by combining OAPM with other clinical phenotypes to provide preliminary prognostic predictions for patients at 1, 3, and 5 years.

### Tumor immune landscape evaluation and prediction of immunotherapy response

First, the relative abundance of 22 immune cell types in the experimental dataset was estimated using the CIBERSORT algorithm. Subsequently, the “ESTIMATE” R package was used to calculate the immune score, stromal score, and ESTIMATE score for OS patients. Additionally, the expression differences of 47 classic immune checkpoint genes were calculated in patients from different risk groups. Finally, based on the expression of the four modeling genes, the patients were divided into two groups, and the transcriptional data of 298 patients with urothelial carcinoma from the IMvigor210 cohort, who received immune therapy, were used to infer differences in immune therapy response between the groups.

### CNV analysis

For the copy number variation (CNV) analysis, the inferCNV algorithm was employed to clarify the CNVs in osteoblasts. Using the infercnv::run function of inferCNV (version 1.2.1), normal osteoblasts were used as a reference for comparison to calculate the changes in gene expression intensity across the chromosomes in the OS cell genome. The parameters were set with denoise=TRUE, analysis_mode=“subcluster,” and a cutoff of 0.1.

### Pseudotime analysis

For the pseudotime analysis in this study, the monocle2 package was utilized. Initially, the newCellDataSet function was used to construct the analysis data for monocle, setting the lowerDetectionLimit to 0.5. Principal component analysis (PCA) was conducted using the plot_pc_variance_explained function, followed by dimension reduction analysis with the reduceDimension function and clustering analysis with the clusterCells function. Subsequently, differential genes were selected to define a cell's progress, and reduceDimension was used to reduce data dimensionality employing the method “DDRTree.” Finally, the orderCells function was used to order cells along the trajectory.

### Cell culture and qRT-PCR experiments

The human osteoblast cell line hFOB1.19 was used as the control cell line, while the OS cell lines 143B, SAOS2, HOS, and U2OS cells were used as the experimental cell lines. All cell lines were purchased from Fuheng Cell Center (Shanghai Fuheng Cell Center, China). The osteoblast cell line was cultured at 33.5 °C and 5% CO_2_ concentration, while the OS cell lines were cultured at 37 °C and 5% CO_2_ concentration. When the cells grew to more than 90% confluency, the supernatant was removed by centrifugation, and the cells were collected for RNA extraction. Total RNA was extracted using the RNA fast 200 Kit (Feijie Biotechnology, China), and cDNA synthesis was performed using the cDNA synthesis kit (Takara, Japan). Subsequently, gene-specific primers were designed and synthesized. The primer sequences are provided in Table 1. The cDNA and primers were mixed in a 10 μl reaction system, with four replicates for each gene. PCR was performed with 10 min at 95 °C, followed by 40 cycles of 10 s at 95 °C and 1 min at 60 °C.

**Table 1:** Primer sequence of the makers gene.

**Gene**	**Primer sequence**
BNIP3-F	CTTCAGCAATAATGGGAACGGG
BNIP3-R	GGTATCTTGTGGTGTCTGCGAGC
SPP1-F	CGAGGTGATAGTGTGGTTTATGG
SPP1-R	GCACCATTCAACTCCTCGCTTTC
MYC-F	GGAAAACCAGCCTCCCGC
MYC-R	CACCGAGTCGTAGTCGAGGT
IGFBP5-F	AAGAAGCTGACCCAGTCCAA
IGFBP5-R	GAATCCTTTGCGGTCACAAT

### Immunohistochemical experiments

To investigate the influence of four model genes on prognosis at the protein level, tumor tissues were collected from six patients with favorable prognosis (survival exceeding 3 years) and six patients with adverse prognosis (survival less than 2 years). The tumor tissue in this immunohistochemical study has been diagnosed as OS by pathologists. After fixation and embedding, sections of 3–5 μm were prepared for immunohistochemical analysis. Dewaxing, antigen retrieval, and hydration were performed, followed by incubation with rabbit anti-BNIP (1:1,000; Beyotime), rabbit anti-IGFBP5 (1:1,000; Beyotime), rabbit anti-SPP1 (1:1,000, Proteintech), and rabbit anti-MYC (1:1,000, Proteintech). After rinsing with PBS, goat anti-mouse/rabbit IgG polymer labeled with enhanced enzyme was added dropwise, and the tissue slices were incubated at 37 °C for 20 min. The staining results were quantitatively analyzed using Image J software. The calculation formula for AOD is AOD (%Area) = IOD/Area.

## Results

### Identification of OS differential genes based on scRNA-seq data

**Figure 2:**
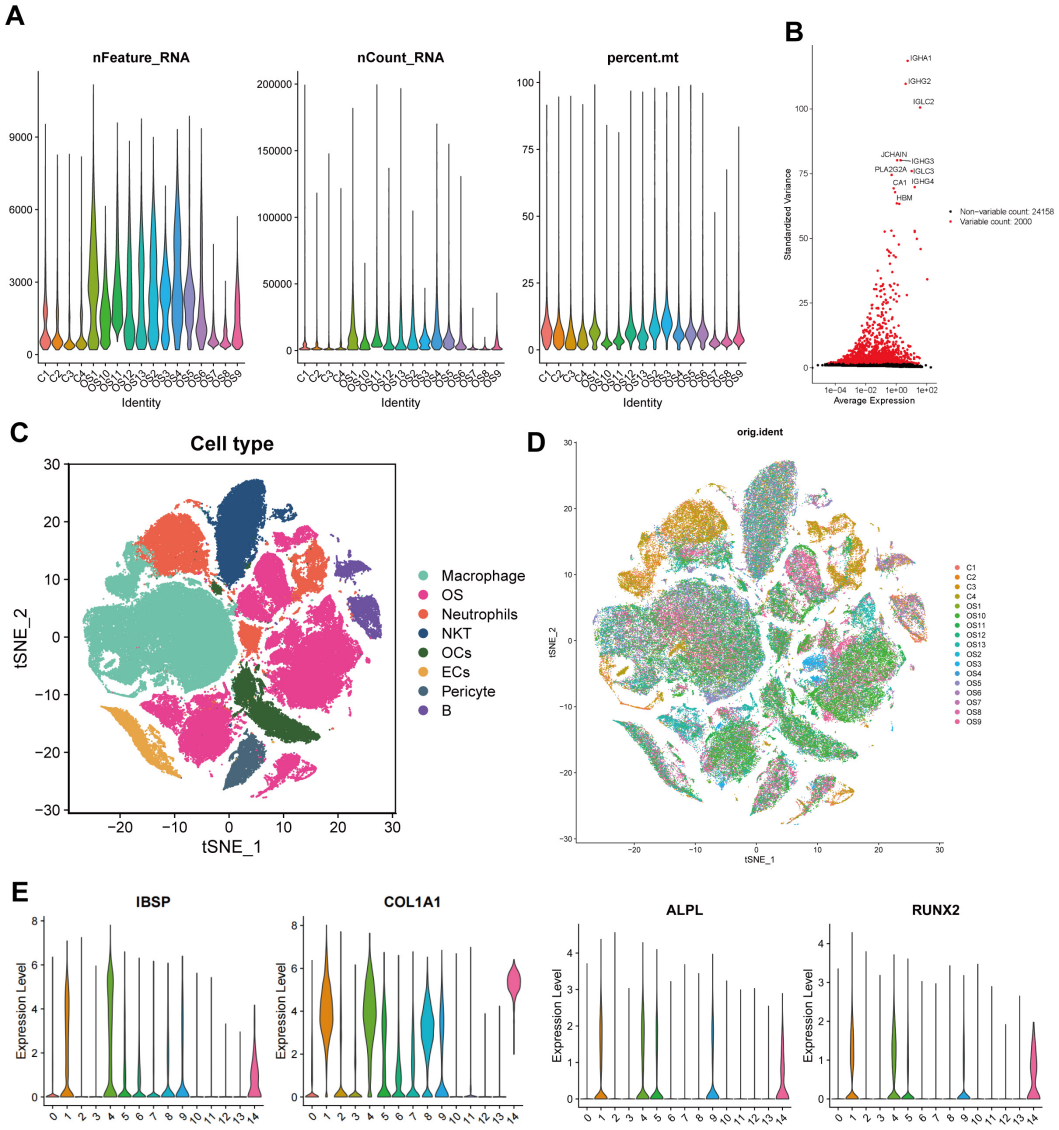
Analysis of single-cell RNA sequencing data. **(A)** Quality control charts for each sample. **(B)** Display of highly variable genes. **(C, D)** tSNE dimensionality reduction and clustering by sample and cluster. **(E)** Marker genes of osteoblasts.

**Figure 3:**
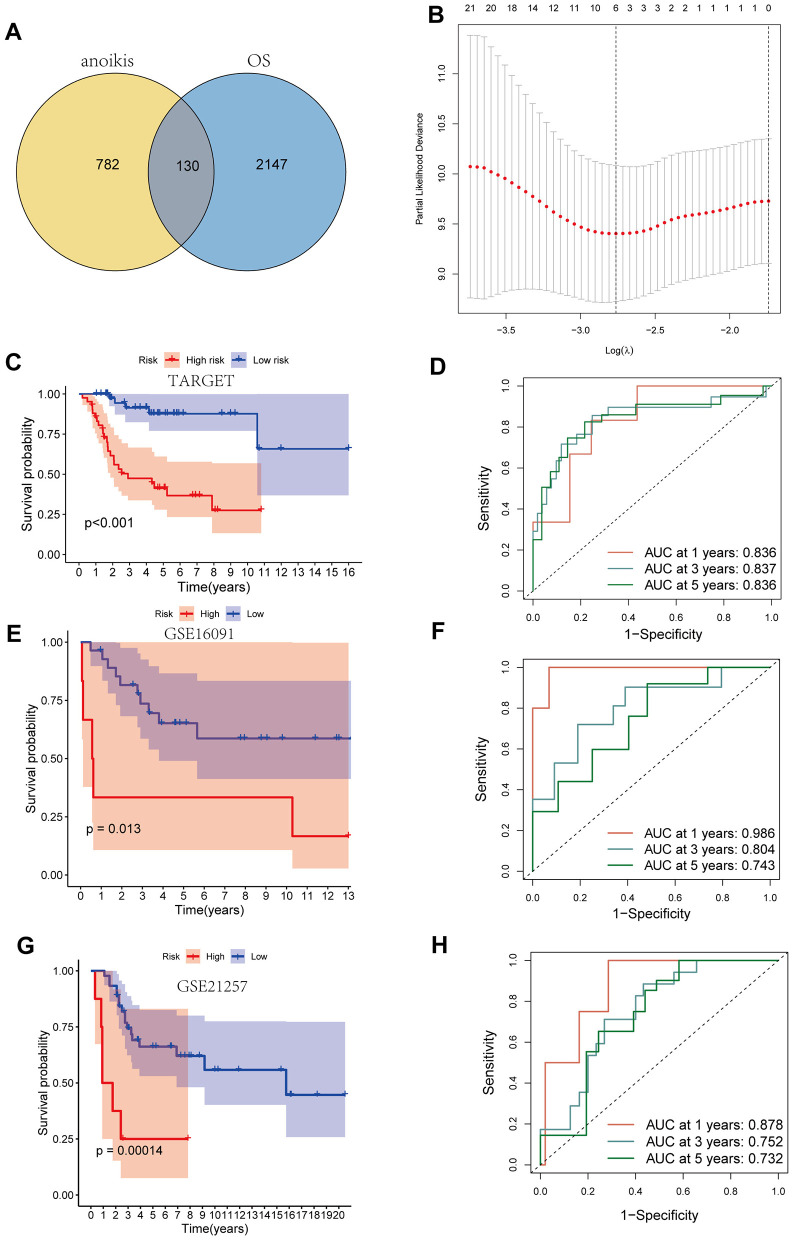
Construction of the prognostic model. **(A)** The venn map of genes related to anoikis and differential genes in osteosarcoma cells. **(B)** LASSO analysis. **(C, D)** Kaplan–Meier curve and ROC curve base on TARGET dataset. **(E, F)** Kaplan-Meier curve and ROC curve base on GSE16091 dataset. **(G, H)** Kaplan–Meier curve and ROC curve base on GSE21257 dataset.

The scRNA-seq data from 13 OS samples and four normal bone tissue samples were integrated and quality-controlled (Figure 2A). PCA was performed using the top 2,000 highly variable genes (Figure 2B). After dimensionality reduction and clustering, a total of 136,140 cells and eight cell subtypes were obtained (Figure 2C). Cells from clusters 1, 4, and 14 predominantly originate from OS tissues and highly express osteoblast markers genes (IBSP, COL1A1, ALPL, and RUNX2). Therefore, 44,896 cells from three clusters have been defined as OS cells (Figures 2D, E). The CNV results indicated that osteoblasts derived from OS tissues exhibited more pronounced copy number variations compared to osteoblasts from normal bone tissues (Supplementary Figure S1). Thus, osteoblasts from normal bone tissue were considered benign, while osteoblast cells from OS tissue were considered malignant, representing OS cells. Subsequently, we compared the gene expression between benign osteoblast cells and OS cells, and a total of 2,277 differential genes were identified (Figure 3A, right circle). These genes were considered to be related to the occurrence, development, and prognosis of OS.

### Construction of the prognostic model

A total of 912 genes related to anoikis were identified using genecards, and the intersection of these genes with the differential genes in OS cells resulted in 130 key genes (Figure 3A). LASSO analysis based on the TARGET database identified four key genes with the greatest impact on prognosis (Figure 3B, Supplementary Figure S2). The final modeling formula was: riskscore = (0.471 ^*^ MYC) + (0.538 ^*^ BNIP3) + (0.320 ^*^ SPP1) + (−0.834 ^*^ IGFBP5). Kaplan–Meier curves showed that patients in the high-risk group had significantly lower survival rates than those in the low-risk group (Figure 3C). The ROC curves indicated that the model had good predictive ability for patient prognosis at 1, 3, and 5 years, with area under the curve (AUC) values of 0.836, 0.837, and 0.836, respectively (Figure 3D). Additionally, in the validation cohort GSE16091, the high-risk group is associated with a poorer prognosis (Figure 3E), with predictive efficiencies for 1, 3, and 5 years of 0.986, 0.804, and 0.743 (Figure 3F), respectively. Similarly, in the validation cohort GSE21257, the high-risk group continues to indicate a poorer prognosis, with very high predictive efficiency (Figures 3G, H). These results demonstrate that our established model can accurately predict the prognosis of OS patients, and the model possesses strong robustness.

**Figure 4:**
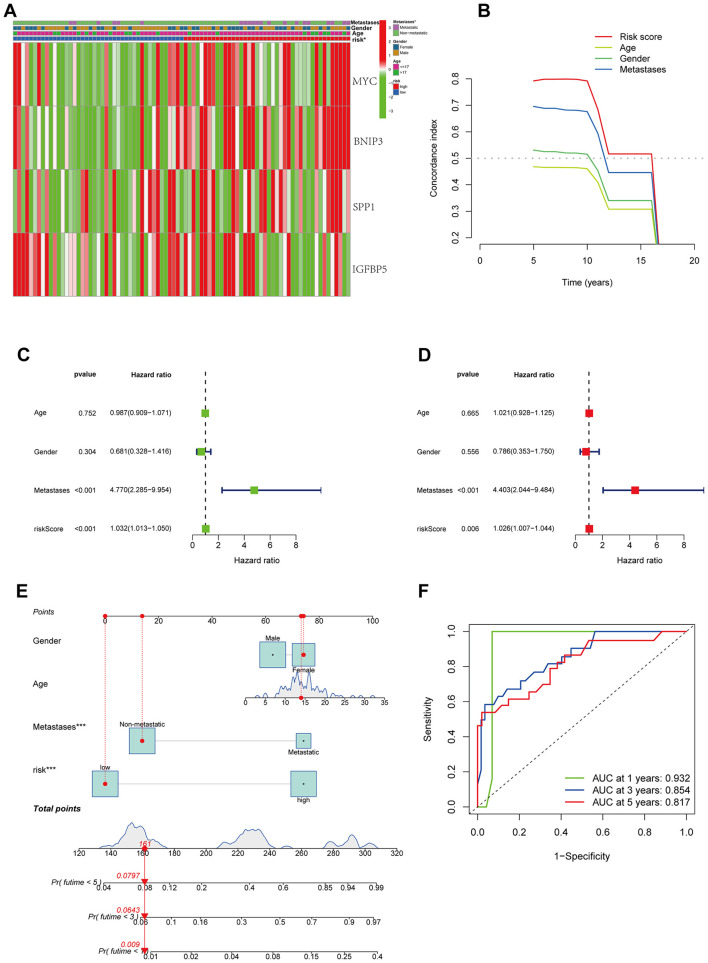
Combination of prognostic models and clinical features. **(A)** The relationship between the modeling genes and clinical characteristics of patients. **(B)** C-index of prognostic models and clinical features. **(C, D)** univariate and multivariate analysis. **(E)** Nomogram of prognostic models and clinical features. **(F)** ROC curve of nomogram.

The relationship between the modeling genes and clinical characteristics of patients was displayed in a heatmap (Figure 4A), showing no significant correlation between the expression of key genes and clinical characteristics, but a clear correlation with the risk score. Furthermore, C-index showed that compared to other clinical characteristics, our model exhibited better predictive performance (Figure 4B). Univariate and multivariate regression analyses indicated that the risk factor could serve as an independent prognostic factor (Figures 4C, D). Subsequently, we combined other clinical features to construct a prognostic forest plot, which showed improved prognostic prediction at 1, 3, and 5 years compared to the risk score alone (Figures 4E, F).

### Pseudotime analysis

**Figure 5:**
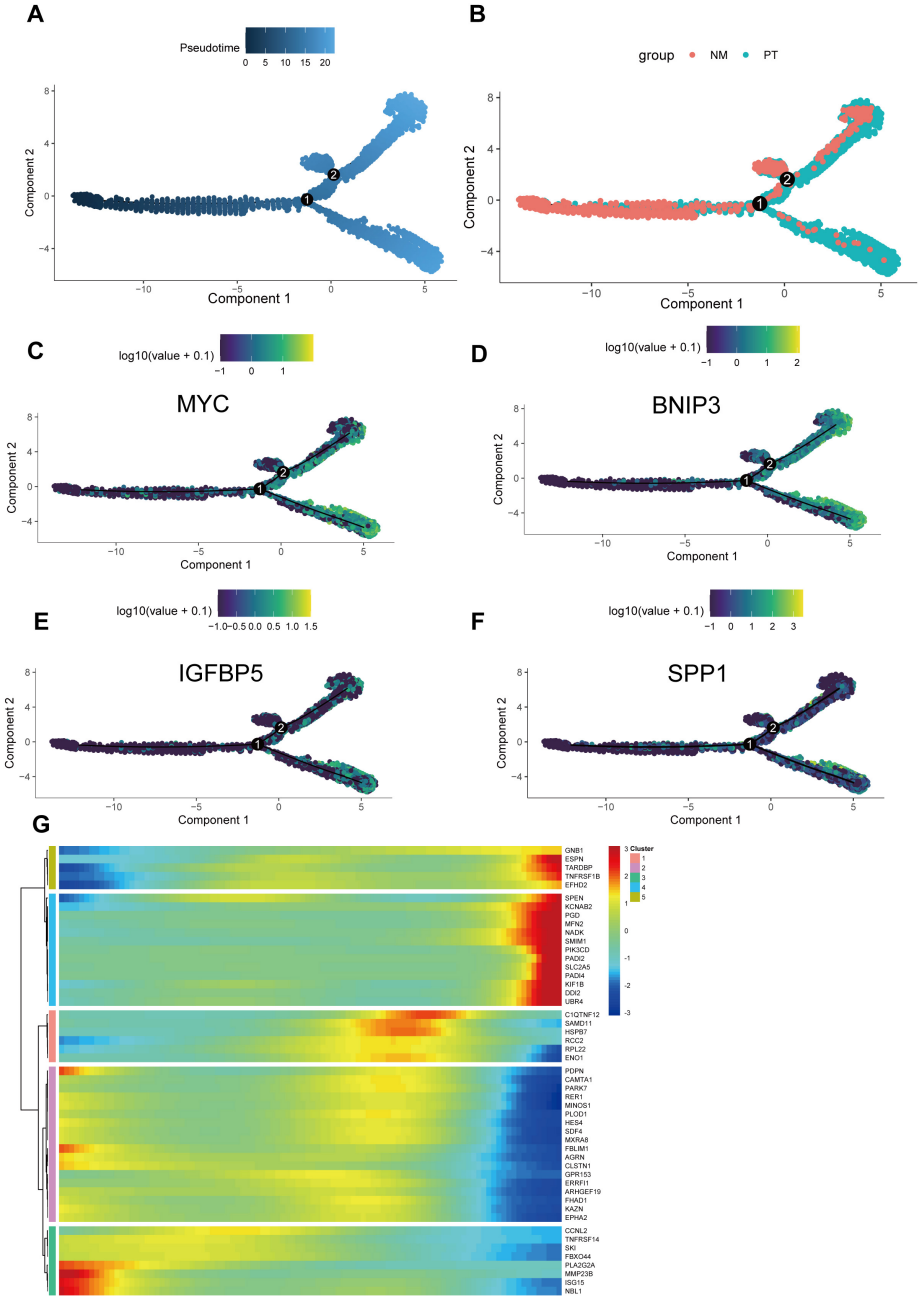
Single-cell trajectory analysis. **(A)** The pseudotime trajectory of cells across two components, with pseudotime values represented by color intensity. **(B)** Two groups: NM (non-metastatic) and PT (primary tumor), color-coded accordingly. **(C–F)** The expression patterns of prognostic models (MYC, BNIP3, IGFBP5, and SPP1) along the pseudotime trajectory, with log-transformed expression values shown. **(G)** Heatmap showing the expression of selected genes across different clusters, with hierarchical clustering applied to both genes and cells. The color bar indicates the level of gene expression in each cluster.

To further explore the developmental trajectories and gene expression changes during the transformation process from normal osteoblasts to OS cells at the single-cell level, we conducted pseudotime analysis. The results indicated that osteoblasts originating from normal tissues were located at the beginning of the pseudotime trajectory, while OS cells derived from OS tissues were positioned at the differentiated end, aligning with the expected trend (Figures 5A, B). The four key genes identified in our model—MYC, BNIP3, SPP1, and IGFBP5—demonstrated a trend of low expression at the beginning and high expression at the end of pseudotime, suggesting their likely involvement in the carcinogenic transition from normal osteoblasts to OS (Figures 5C–F). This provided a theoretical foundation for our model. Additionally, we identified another 50 genes whose expression changes along the pseudotime; these genes may participate in the pathogenesis of OS and warrant further research attention (Figure 5G).

### Relationship between the risk model and immune cell infiltration

**Figure 6:**
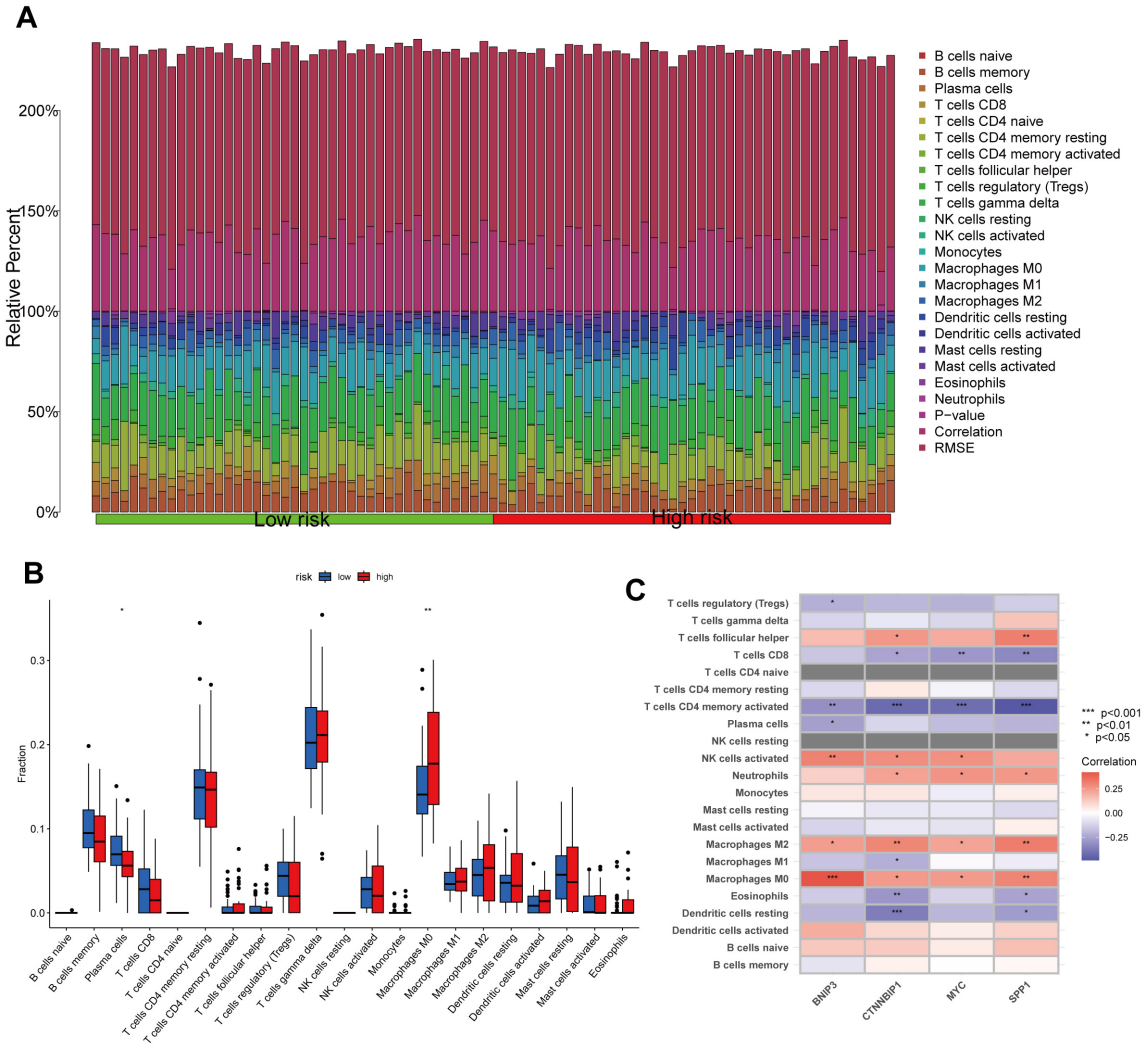
Relationship between the risk model and immune cell infiltration. **(A, B)** Histogram and boxplot of immune cell infiltration. **(C)** Correlation analysis between immune cell and modeling genes. *p < 0.05, **p < 0.01, ***p < 0.001.

**Figure 7:**
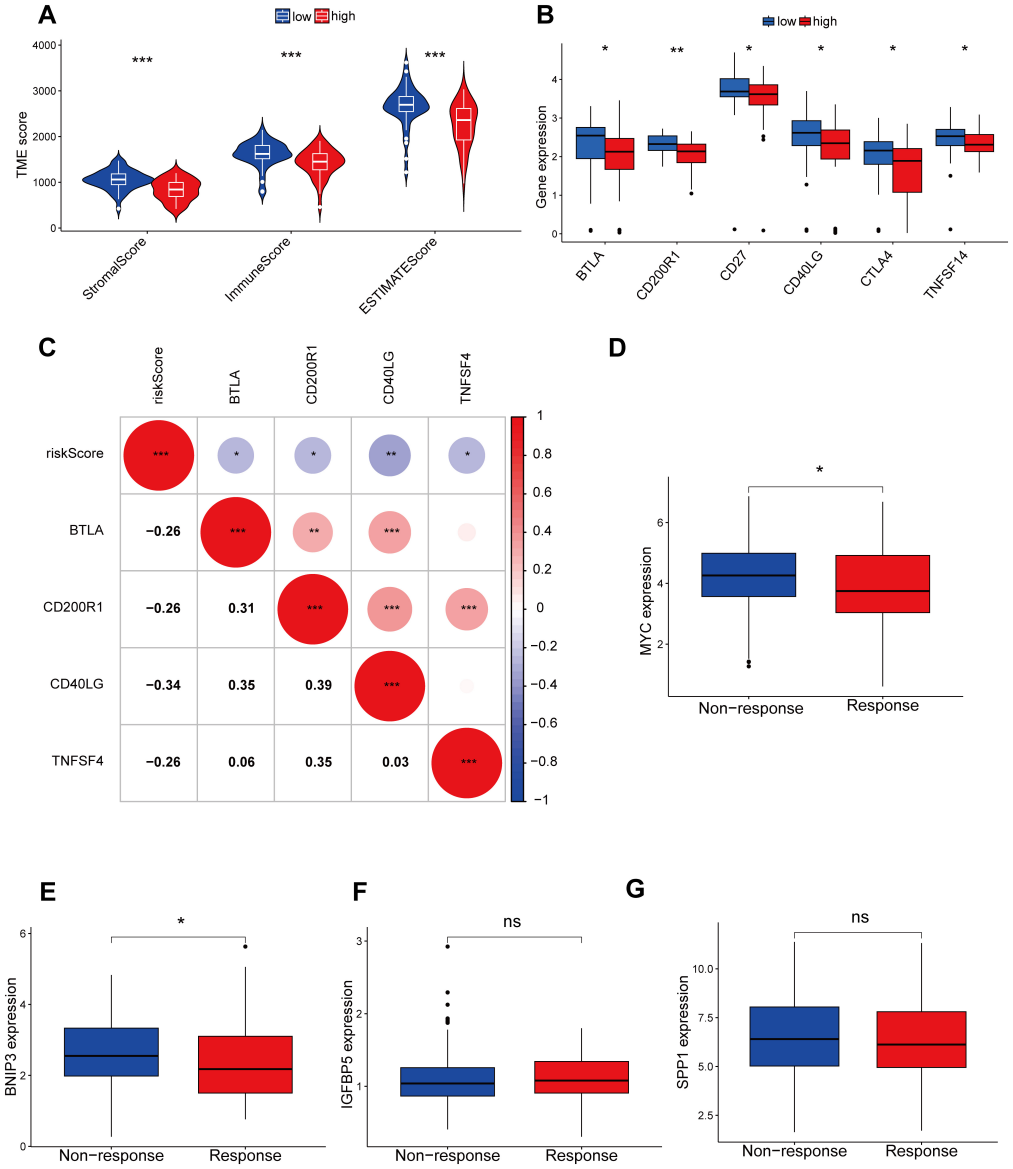
Immunological checkpoints and immunotherapy analysis. **(A)** The ESTIMATE algorithm of immune cell infiltration. **(B)** Differences in immune checkpoint between high and low risk groups. **(C)** The correlation between risk models and immune detection points. **(D–G)** The correlation between model genes and immune therapy responses. *P < 0.05, **P < 0.01, ***P < 0.001, ns, not significant.

The CIBERSORT algorithm results showed that the low-risk group had higher abundance of plasma cells, while the high-risk group had higher abundance of macrophages M0, indicating that these cells may be involved in the immune regulation between different risk groups (Figures 6A, B). Additionally, the modeling hub genes were associated with many immune checkpoint genes, suggesting their potential relevance to immune therapy in OS (Figure 6C). The ESTIMATE algorithm revealed that the low-risk group had higher immune infiltration levels (Figure 7A), and the low-risk group exhibited more immune therapy checkpoint gene expressions (Figures 7B, C), indicating that the low-risk group may benefit more from immune therapy. Importantly, BNIP3 and MYC were all associated with the response to immune therapy, further suggesting that our model may be applicable to predicting clinical responses to immune therapy (Figures 7D–G).

### qRT-PCR and ICH results

**Figure 8:**
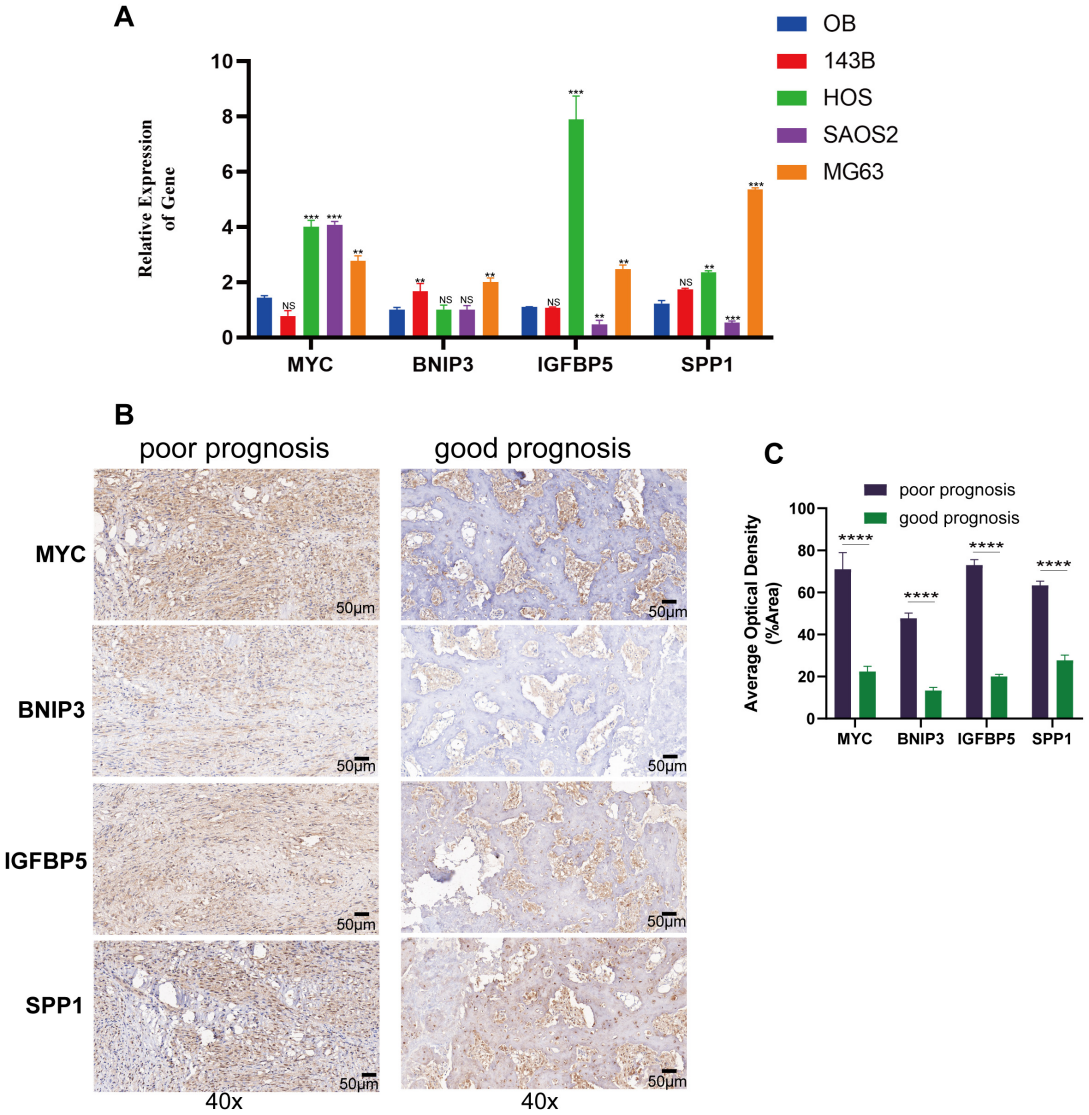
Gene expression and immunohistochemistry analysis of modeling genes in osteosarcoma cell lines and tissues. **(A)** The qRT-PCR results of human osteoblast cell and osteosarcoma cell lines. **(B)** Immunohistochemical staining of MYC, BNIP3, IGFBP5, and SPP1 in tumor tissues from patients with poor prognosis (left) and good prognosis (right) at 40× magnification. **(C)** Quantification of average optical density for MYC, BNIP3, IGFBP5, and SPP1 staining in tumor tissues from poor and good prognosis patients. **p < 0.01, ***p < 0.001, ****P < 0.0001, ns = not significant.

The qRT-PCR results showed that compared to normal osteoblast cells, MYC, BNIP3, IGFBP5, and SPP1 substantially exhibited a trend of high expression (Figure 8A). The results of the immunohistochemistry suggested that in OS patients with poorer prognosis, the expression of these four hub genes was significantly elevated (Figures 8B, C). These results further confirmed the key roles of the four hub genes on the carcinogenic and prognosis in OS.

## Discussion

OS is a highly malignant primary bone tumor with a poor prognosis. Anoikis resistance, a form of programmed cell death, is crucial for the survival of detached tumor cells from the extracellular matrix. Resistance to anoikis has been identified as a key factor in tumor progression in invasive tumor cells (7, 8), yet there's limited research on the relationship between OS and anoikis. Additionally, previous prognostic models for OS cell death were based on Bulk RNA sequencing, potentially neglecting the tumor microenvironment's impact on prognosis. In this study, we started with single-cell RNA data to identify and extract OS cells. By combining specific markers of OS cells with anoikis, we identified key genes. Subsequently, we modeled and predicted immune infiltration using Bulk RNA sequencing data.

Our prognostic model comprises four key genes: MYC, BNIP3, IGFBP5, and SPP1. The Bcl-2/adenovirus E1B 19 kDa interacting protein 3 (BNIP3) gene encodes a mitochondrial protein containing the BH3 domain, which functions in promoting apoptosis and mitochondrial autophagy (9). Although BNIP3 is often considered a protective gene that inhibits cancer growth, studies have shown that inhibiting BNIP3 gene expression leads to breast cancer progression. IGFBP5 is a secreted protein belonging to the IGFBP (Insulin-like Growth Factor Binding Protein) family, primarily regulating the specific binding between Insulin-like Growth Factors (IGFs) and IGF receptors. IGFBP5 has been demonstrated to be associated with the malignant progression of many cancers. For instance, in glioblastoma, IGFBP5 can promote tumor invasion through the ROR1/HER2-CREB signaling pathway (10, 11). MYC is a transcription factor and a classic oncogene, with mutations closely associated with the development of various cancers. Myc can also prevent immune cells from effectively attacking tumor cells, aiding in the tumor cell's immune evasion process (12). Phosphoprotein 1 (SPP1), also known as osteopontin, is a multifunctional, secreted phosphorylated glycoprotein. At the cellular level, SPP1 expression is limited to a few cell types such as osteoblasts, fibroblasts, macrophages, dendritic cells, lymphoid cells, and mononuclear cells of the immune system. SPP1 is also expressed by cancer cells. Previous studies have established correlations between elevated levels of circulating SPP1 or increased expression of SPP1 on tumor cells and poor prognosis in many types of cancer. SPP1 plays a significant role in promoting cancer cell growth and resistance to chemoradiotherapy by inducing epithelial-mesenchymal transition, autophagy, aberrant glucose metabolism, epigenetic changes, and reducing drug uptake (13). Overall, our model genes are closely related to cancer, suggesting their potential involvement in OS development.

The development of a prognostic model for OS has been a key focus in OS treatment. Some studies have used scRNA-seq to identify tumor-associated lymphocytes and construct prognostic models based on these markers (14). Others have utilized ARGs to build prognostic models for various tumors (15). However, there have been limited studies on modeling OS using a combination of scRNA-seq and ARGs. In our study, we first incorporated scRNA-seq data from six OS samples and four normal bone samples to identify osteoblasts. We then calculated differentially expressed genes between the two groups of osteoblasts. Subsequently, we intersected these DEGs with key ARGs to identify crucial genes and construct a robust prognostic prediction model. Our model integrates scRNA-seq data and considers the tumor microenvironment, offering a potentially more accurate and reliable approach for OS prognosis prediction.

The theory of the tumor microenvironment suggests that a tumor is composed not only of tumor cells but also of immune cells, endothelial cells, fibroblasts, and other components (16). Among these, the tumor immune microenvironment plays a critical role in tumor development and treatment (17). In recent years, immunotherapy has achieved significant success in cancer treatment, fundamentally changing the outcome of cancer therapy (18). Unfortunately, the application of immunotherapy in OS remains limited. In our study, we utilized the expression levels of model genes to classify patients from the TARGET database into high and low groups, determining the degree of immune cell infiltration between these groups. We also explored the relationship between the four model genes and the response to immunotherapy. This information may provide a basis and guidance for immunotherapy strategies in OS treatment.

In conclusion, we have developed an effective model for predicting OS prognosis and immune response. This model could serve as a valuable reference for OS prognosis assessment and the design of immunotherapy strategies.

## Data Availability

The original contributions presented in the study are included in the article/Supplementary material, further inquiries can be directed to the corresponding authors.

## Supplementary material

Supplementary Figure S1 inferCNV analysis showing the copy number variation (CNV) landscape across osteosarcoma cells.

Supplementary Figure S2 Bar chart illustrating the significance levels of multiple testing correction for modeling genes (MYC, BNIP3, SPP1, and IGFBP5). The hazard ratios (HR) for each gene are shown alongside the significance levels. Genes with a Bonferroni < 0.05 are marked in red, indicating statistical significance after Bonferroni correction. Genes with an FDR < 0.05 are shown in orange, indicating statistical significance after FDR correction. Genes that were not significant are shown in gray.

## References

[1] Beird HC Bielack SS Flanagan AM Gill J Heymann D Janeway KA . Osteosarcoma. Nat Rev Dis Primers. (2022) 8:77. doi: 10.1038/s41572-022-00409-y 36481668

[2] Pan R Pan F Zeng Z Lei S Yang Y Yang Y . A novel immune cell signature for predicting osteosarcoma prognosis and guiding therapy. Front Immunol. (2022) 13:1017120. doi: 10.3389/fimmu.2022.1017120 36189307 PMC9515362

[3] Tajbakhsh A Rivandi M Abedini S Pasdar A Sahebkar A. Regulators and mechanisms of anoikis in triple-negative breast cancer (TNBC): a review. Crit Rev Oncol Hematol. (2019) 140:17–27. doi: 10.1016/j.critrevonc.2019.05.009 31154235

[4] Wang J Luo Z Lin L Sui X Yu L Xu C . Anoikis-associated lung cancer metastasis: mechanisms and therapies. Cancers. (2022) 14:4791. doi: 10.3390/cancers14194791 36230714 PMC9564242

[5] Raeisi M Zehtabi M Velaei K Fayyazpour P Aghaei N Mehdizadeh A. Anoikis in cancer: the role of lipid signaling. Cell Biol Int. (2022) 46:1717–28. doi: 10.1002/cbin.11896 36030535

[6] Sun Z Zhao Y Wei Y Ding X Tan C Wang C. Identification and validation of an anoikis-associated gene signature to predict clinical character, stemness, IDH mutation, and immune filtration in glioblastoma. Front Immunol. (2022) 13:939523. doi: 10.3389/fimmu.2022.939523 36091049 PMC9452727

[7] Dai Y Zhang X Ou Y Zou L Zhang D Yang Q . Anoikis resistance–protagonists of breast cancer cells survive and metastasize after ECM detachment. Cell Commun Signal. (2023) 21:190. doi: 10.1186/s12964-023-01183-4 37537585 PMC10399053

[8] Taddei M Giannoni E Fiaschi T Chiarugi P. Anoikis: an emerging hallmark in health and diseases. J Pathol. (2012) 226:380–93. doi: 10.1002/path.3000 21953325

[9] Fu ZJ Wang ZY Xu L Chen XH Li XX Liao WT . HIF-1α-BNIP3-mediated mitophagy in tubular cells protects against renal ischemia/reperfusion injury. Redox Biol. (2020) 36:101671. doi: 10.1016/j.redox.2020.101671 32829253 PMC7452120

[10] Lin W Niu R Park SM Zou Y Kim SS Xia X . IGFBP5 is an ROR1 ligand promoting glioblastoma invasion via ROR1/HER2-CREB signaling axis. Nat Commun. (2023)14:1578. doi: 10.1038/s41467-023-37306-1 36949068 PMC10033905

[11] Wang H Rosen DG Wang H Fuller GN Zhang W Liu J. Insulin-like growth factor-binding protein 2 and 5 are differentially regulated in ovarian cancer of different histologic types. Mod Pathol. (2006) 19:1149–56. doi: 10.1038/modpathol.3800637 16729015

[12] Casey SC Tong L Li Y Do R Walz S Fitzgerald KN . MYC regulates the antitumor immune response through CD47 and PD-L1. Science. (2016) 352:227–31. doi: 10.1126/science.aac9935 26966191 PMC4940030

[13] Matsubara E Yano H Pan C Komohara Y Fujiwara Y Zhao S . The significance of SPP1 in lung cancers and its impact as a marker for protumor tumor-associated macrophages. Cancers. (2023) 15:2250. doi: 10.3390/cancers15082250 37190178 PMC10136569

[14] Tang H Liu S Luo X Sun Y Li X Luo K . A novel molecular signature for predicting prognosis and immunotherapy response in osteosarcoma based on tumor-infiltrating cell marker genes. Front Immunol. (2023) 14:1150588. doi: 10.3389/fimmu.2023.1150588 37090691 PMC10117669

[15] Chen S Gu J Zhang Q Hu Y Ge Y. Development of biomarker signatures associated with anoikis to predict prognosis in endometrial carcinoma patients. J Oncol. (2021) 2021:3375297. doi: 10.1155/2021/3375297 34992654 PMC8727165

[16] Ren X Zhang L Zhang Y Li Z Siemers N Zhang Z. Insights gained from single-cell analysis of immune cells in the tumor microenvironment. Annu Rev Immunol. (2021) 39:583–609. doi: 10.1146/annurev-immunol-110519-071134 33637019

[17] Lei X Lei Y Li J Du W Li R Yang J. Immune cells within the tumor microenvironment: biological functions and roles in cancer immunotherapy. Cancer Lett. (2020) 470:126–33. doi: 10.1016/j.canlet.2019.11.009 31730903

[18] Kennedy L Salama A. A review of cancer immunotherapy toxicity. CA Cancer J Clin. (2020) 70:86–104. doi: 10.3322/caac.21596 31944278

