# Supplementary figures and images for: Prognostic value and immune infiltration of anoikis-related genes in osteosarcoma
Source: Front Med (Lausanne). 2025 Nov 6;12:1669470. doi: 10.3389/fmed.2025.1669470 (PMC12631436; doi:10.3389/fmed.2025.1669470)

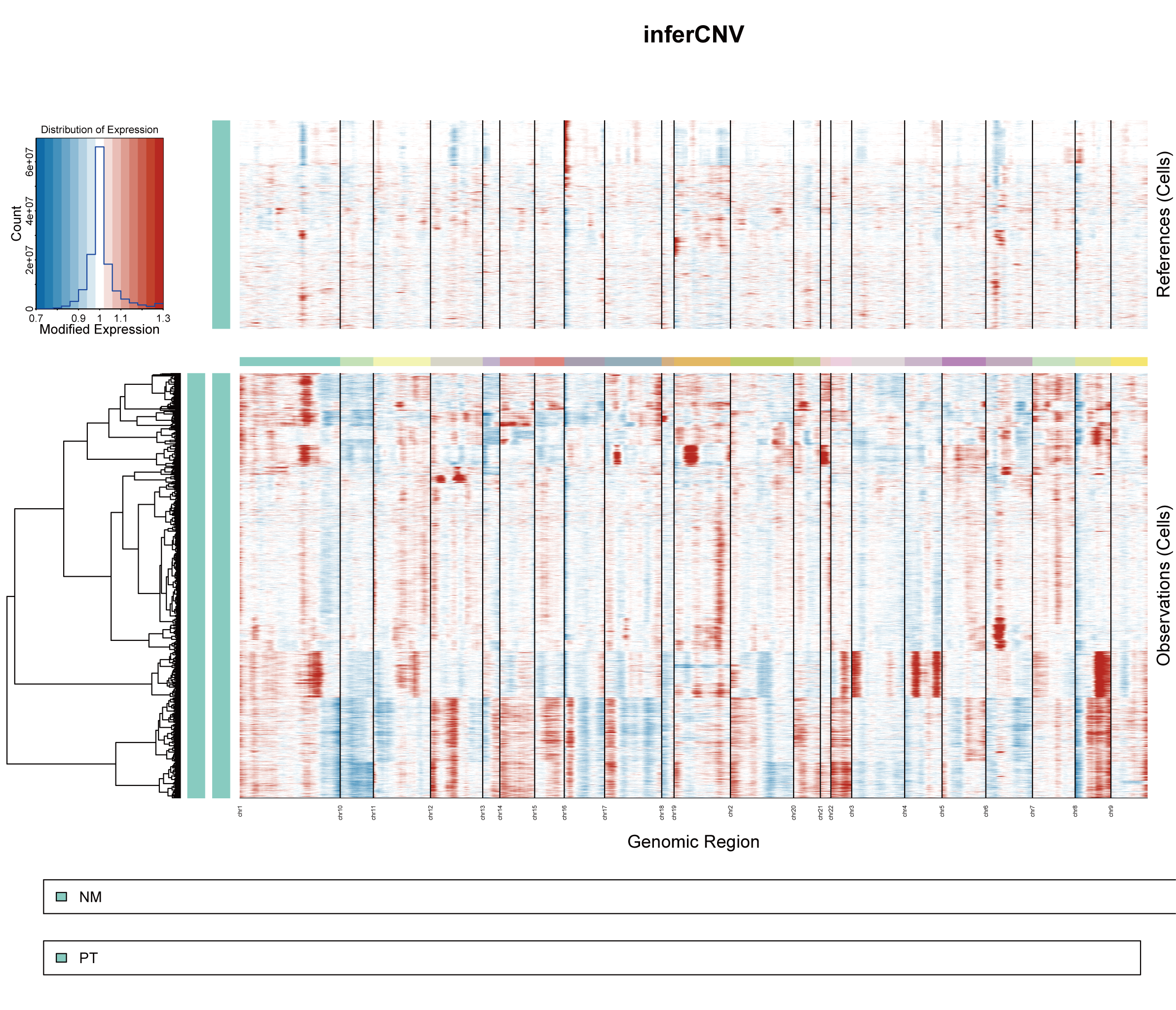

Supplement: Supplementary Figure S1 — inferCNV analysis showing the copy number variation (CNV) landscape across osteosarcoma cells. [file Image_1.tif]

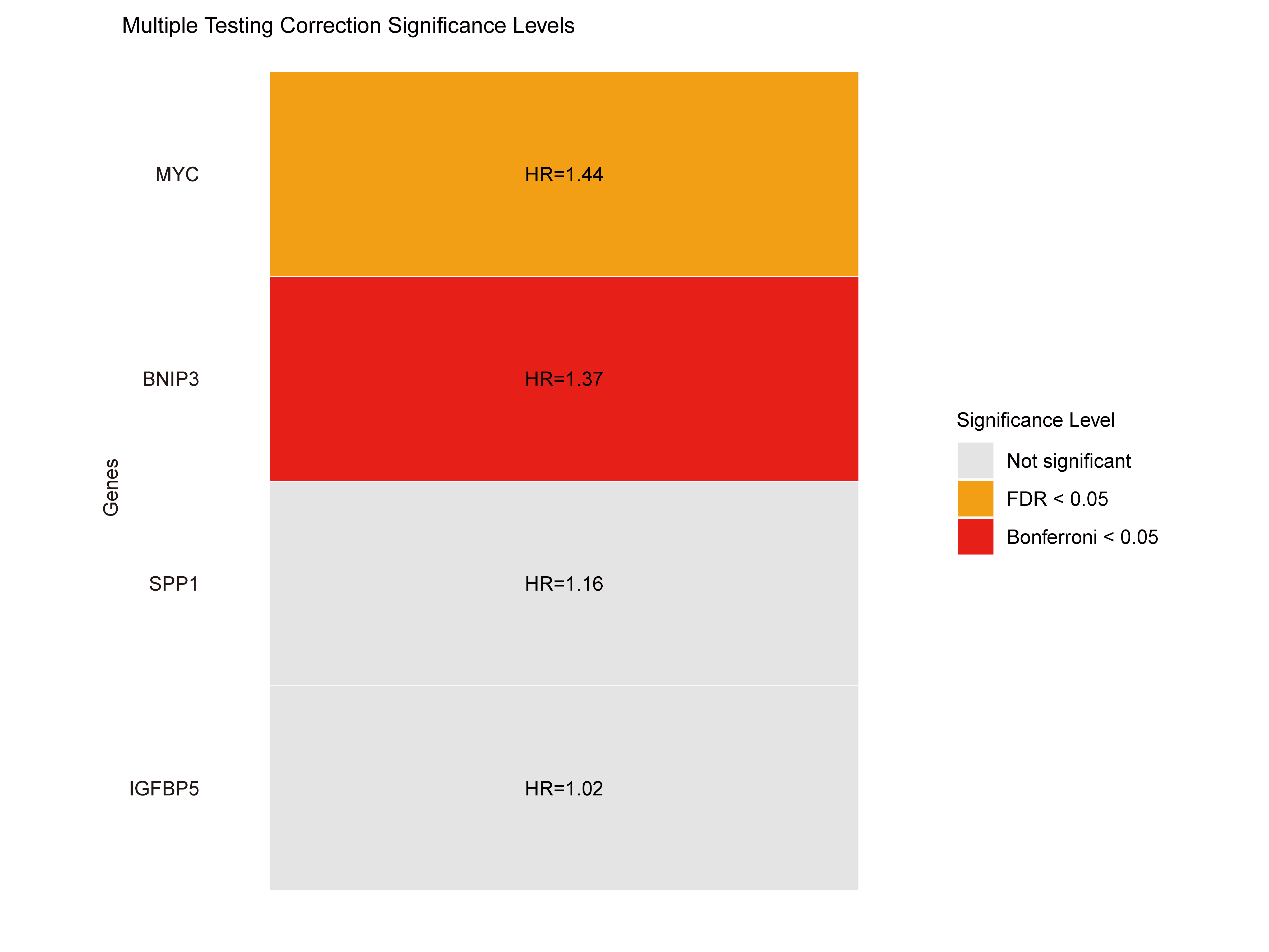

Supplement: Supplementary Figure S2 — Bar chart illustrating the significance levels of multiple testing correction for modeling genes (MYC, BNIP3, SPP1, and IGFBP5). The hazard ratios (HR) for each gene are shown alongside the significance levels. Genes with a Bonferroni < 0.05 are marked in red, indicating statistical significance after Bonferroni correction. Genes with an FDR < 0.05 are shown in orange, indicating statistical significance after FDR correction. Genes that were not significant are shown in gray. [file Image_2.tif]
